# Supplementary material for: Are carbon emissions trading and green financial instruments synergistic? -Comprehensive quantitative research based on content analysis
Source: PLoS One. 2024 Mar 7;19(3):e0298601. doi: 10.1371/journal.pone.0298601 (PMC10919601; doi:10.1371/journal.pone.0298601)
Supplement: S1 Data — (ZIP) [file pone.0298601.s001.zip › Data/地级市Z_F工作报告.zip!/╜¡╬≈/╥╦┤║╩╨/└┤╫╘╣1⁄2╓┌║┼í╛┬φ┐╦╩2╛▌═°í┐_2010.docx]

各位代表：现在，我代表市人民政府向大会作政府工作报告，请予审议，并请各位市政协委员和列席会议的同志提出意见。

一、2009年工作简要回顾

2009年，全市人民在省委、省政府和市委的坚强领导下，按照“三保一弘扬”的要求，围绕“止后移、争进位、站前列”的目标，以开展深入学习实践科学发展观活动为动力，解放思想，积极作为，共克时艰，完成了市二届人大四次会议确定的主要任务。

———最令人鼓舞的是，我们在应对金融危机中保持了经济快速增长。预计全年实现生产总值705亿元，增长13%；财政总收入80.61亿元，增长24.2%，地方财政收入47.66亿元，增长 30.7%；规模以上工业增加值 247.93亿元，增长 20.5%；全社会固定资产投资512亿元，增长 40.1%；社会消费品零售总额226.1亿元，增长18.7%；实际利用外资 3.26亿美元，增长 11.6%；引进省外5000万元以上工业项目资金 135.1亿元，增长26.1%；外贸出口 3.29亿美元，增长 15.7%；城镇居民人均可支配收入 1.3万元，增长 8.9%，农民人均纯收入 5100元，增长 9%。部分经济指标增幅在全省排位前移或进入全省前列。县域经济实力明显增强，丰城财政收入突破 20亿元大关，樟树超 12亿元，高安超 8亿元，袁州和上高分别超 6亿元，万载和奉新分别超 5亿元，财政总收入过亿元乡镇（街道）达到 5个。全市经济结构及增长质量不断优化。三次产业占GDP的比重由上年的 21.7 52 26.3调整为 20.6 53.9 25.5；财政总收入占GDP的比重达11.4%，同比提高 0.8个百分点。民营经济主力军地位更加突出，全年实现税收 48亿元，占全市税收收入比重达到66.4%。

———最令人振奋的是，我们在实施产业建设上取得了重大项目突破。全年共引进项目 352个，其中投资亿元以上、10亿元以上的重大项目分别达81个、12个。预计十大产业基地290家生产企业共完成主营业务收入 320亿元，增长 35%，实现利税 45亿元，增长 30%。宜春中心城锂电新能源、医药、建材、机电、服务业、油茶等六大产业强势推进，特别是锂电新能源产业列入全省第四个千亿元工程和省重点发展产业，建立了全国第一个锂电产业园，并被批准为“国家锂电新能源高新技术产业化基地”；丰城富硒产业基地获“中国生态硒谷”称号、赣中再生金属集散市场被列为全国第二批再生资源回收体系建设单位；奉新纺织基地被授予“中国新兴纺织产业基地”。丰城华伍制动工业制动器、袁州飓风化工乙撑胺、济民可信金水宝系列产品等项目被列为省政府高新产业重大项目。

———最令人欣喜的是，我们在推进民生工程中实现了总体水平提升。民生支出97.9亿元，增长 34.1%，占财政支出比重达 70.7%，高出财政支出增幅 1.7个百分点。民生工程 8大方面、91项指标全面完成目标任务，其中超额完成目标任务的 61项，占 67%。全市38.1万返乡农民工实现就业创业36.9万人，跨省劳务输出总数达73.6万人，恢复并超过金融危机前水平，中央电视台焦点访谈、新华社等主流媒体对我市帮助返乡农民工就业创业情况进行了专题报道。“光明·微笑”工程成效居全省前列。

回顾过去的一年，我们主要抓了以下工作：（一）着力把“抓项目”作为保持经济增长的重点。抢抓国家扩大内需的政策机遇，全力跑项争资，争取国家和省项目资金 18.62亿元，同比增长 50%，占全省的 10.5%。在中央已下达的四批扩大内需项目资金中，我市有 949个项目获得国家支持，共争取中央预算内资金 10.59亿元。重大项目建设进展顺利，宜春明月山机场“三通一平”工程全面展开，靖安洪屏抽水蓄能电站通过国家发改委审批，昌铜高速公路南昌—奉新（靖安）段已开工建设，奉新（靖安）—铜鼓段完成初步设计方案，杭长铁路客运专线已确定初步设计方案，高安建筑陶瓷产业基地、樟树新干盐化工基地铁路专用线全面开工建设，高胡一级公路竣工通车，咸宜吉铁路、宜春铁路货场、丰电三期火力发电项目前期工作进展良好。从 5月下旬开始，先后开展了“产业招商百日大会战”和“项目落地百日大会战”，中心城区组织了“开工建设六十项目，喜迎国庆六十周年”活动。樟树宏宇化工和四特酒科技工业城、丰城新高焦化、上高东新鞋业、高安罗斯福陶瓷、奉新新源纺织、市本级兴发铝业等一大批重大项目纷纷落户。围绕创建最优发展环境，开辟了重大项目审批服务“绿色通道”，在工业园区全面实行“六个严禁”；认真落实中央和省减轻企业负担的各项政策，金融危机发生以来，共减免缓税 5.81亿元；新增中小企业信用担保机构 8家，高安市中小企业信用担保中心成为全省首个注册资本过亿元的县级信用担保机构；全市金融机构信贷投放量同比翻番，赣州银行宜春分行成功进驻。

（二）着力把“促繁荣”作为加快城市建设的重点。城镇化率达 40%，同比提高 1.8个百分点。宜居城市创建达到上榜城市标准，中心城区城市建设管理暗访连续 5年排名全省第一，新增全国社会治安综合治理优秀市、国家现代林业建设示范市、世界著名文化旅游城市、中国旅游竞争力百强城市、中国最具民俗文化特色旅游目的地 5张城市名片。万载获省级卫生城称号，丰城通过省级园林城市评审，靖安被命名为首批省级生态县。樟树、上高、丰城、高安污水处理设施投入运行，其余各县进入试运行阶段，中心城区污水处理一期续建工程投入运行，污水处理能力达 8万吨 /日，污水处理率达 80%以上。主要污染物减排成果突出，二氧化硫减排提前一年超额完成十一五规划目标，化学需氧量削减 2684吨。闲置土地清理初见成效，清理出各类闲置土地 1.7万余亩。“三大战役”加速向纵深推进：工业强攻战成效显著，宜春经济开发区引进 10亿元以上项目 6个；城市扩张战成果突出，已实施城建项目67个，总投资达 40亿元；旅游升温战战果丰硕，明月山温泉风景名胜区接待游客 75万人次，增长 25%，旅游总收入 5亿元，增长32%。中心城区亮化改造工程开始启动。“13579”绿色工程顺利推进，完成投资 1.7亿元，新增绿地面积50.76万平方米，改造绿地面积 20.42万平方米。人口倍增计划成效明显，中心城区总人口达 45万。通过深入开展文明社区、文明城市创建活动，市民的文明素质进一步提高。成功举办了第三届月亮文化节暨经贸活动周、全国第40届樟树药交会，尤其是“宜春月·中华情”央视中秋晚会获得海内外高度赞誉。万载县成功举办首届海峡两岸花炮产业发展高峰论坛。

（三）着力把“强产业”作为提升“三农”水平的重点。惠农政策落实到位，共发放各类补贴资金 6.6亿元。粮食生产连续六年增产，樟树被评为全国粮食生产先进县（市）标兵，宜丰被列为全国高标准农田建设示范县。生猪出栏 596万头，农业综合机械化水平突破40%，均创历史最高水平。农业招商取得突破，共引进农业项目 155个，其中亿元以上 26个。农业产业化水平进一步提高，初步形成了粮食生产加工、中药材加工、禽业加工、木竹加工、油茶加工等五大产业聚集区，万载、宜丰、铜鼓、靖安的有机农产品和丰城的有机富硒农产品逐步成为产业化新亮点。市级以上龙头企业增加到 168家，完成销售收入 161.5亿元，增长 23.2%。农民组织化程度不断提高，全市注册专业合作社达 705家，成员 3.8万人。阳光工程完成培训农民 3.2万人。新建农村户用沼气池12814个。新农村建设水平提高，880个省定村点均制定产业发展规划，发展“一村一品”产业969个，改水入户率达 99.95%，改厕入户率达99.79%，改路 1326公里，建设科技文化、体育休闲、医疗卫生等五类设施 3546个。造林绿化“一大四小”工程建设超额完成任务，上高、奉新、丰城、高安被评为全省造林绿化“一大四小”工程建设先进县（市）。农村清洁工程全面启动，5573个自然村、59个集镇及其范围内的学校、卫生院所纳入试点范围，共建垃圾焚烧炉1566座、沼气池 6274个，处理垃圾 8.43万吨。病险水库治理累计完成投资 4.2亿元。9.61万人安全饮水问题得到解决。农技推广体系建设加快，万载、靖安、奉新、袁州被列为全省试点县，基层农技站改革工作全面开展。

（四）着力把“活机制”作为增强内生动力的重点。大力推进国有企业改革。全市1424家国有集体企业，已改制和正在改制1227家，改制面达90%；238家国有工业企业的改革已进入扫尾阶段，其中7家市直工业企业除2家正在扫尾外，其余均已完成改制。政府机构改革稳步推进，95%以上的事业单位完成岗位设置。稳妥推进医药卫生体制改革，按照“1个贯彻意见+4个实施方案”的形式，市委、市政府下发了《关于深化医药卫生体制改革的贯彻意见》。巩固深化行政审批制度“两集中、两到位”改革，市县两级同步改革、同步到位，走在全省前列。深化财政各项改革，继续推进部门预算改革，大力推进县级部门预算改革试点，县级执收执罚部门收支完全脱钩试点县扩大到6个。国有农场税费改革基本完成，上级财政转移支付率达100%，“两田制”改革全部执行到位，办社会职能有效分离。“普九”化债工作稳步推进，全市累计化债6.6亿元，占债务总额的98.7%。国库集中收付制度改革扩大到7个县市区，公务卡改革稳步实施。加强财政基建评审工作，积极推进政府采购“管采分离”，采购规模和范围进一步扩大。全市政府采购规模达6.2亿元，节约资金7000万元。稳步推进企业上市，按照“上市一批、储备一批、辅导一批”的原则，制定了上市规划，全市后备资源库企业达28家。丰城华伍制动完成上市辅导，已向国家证监委申报发行材料；高安正邦化工进入辅导期；袁州百神药业进入股改阶段；袁州远大化工与中介机构达成协议，启动股改计划；袁州金特建材等企业的上市前期工作进展顺利。

（五）着力把“惠民生”作为和谐平安建设的重点。全面启动了创建首批国家级创业型城市工作，通过对外输出、就近转移、创业带动、培训帮助、园区吸纳，使农民工返乡潮变创业潮，全市城镇新增就业人数8.98万人，新增转移农村劳动力17.76万人。“光明·微笑”工程提前超额完成任务，为1.3683万名白内障和742名唇腭裂患者成功实施手术。为义务教育阶段学生全部免除学杂费、免费提供教科书，新建、改扩建校舍面积17.57万平方米。开工建设经济适用住房6.9万平方米、990套，新建廉租住房28.15万平方米、5630套。全市28.8万人领取城乡低保金，城乡最低生活保障标准分别达到222元和104元，月人均补助水平分别达到160元和66元，为27.2万人次实施医疗救助，发放医疗救助资金9606万元，为1138户农村困难群众新建维修住房。城镇职工参加基本养老保险人数52.8万人，参加基本医疗保险人数65.4万人，覆盖率为92%，城镇居民基本医疗保险覆盖率达100%，新农合参保农民达到376.3万人，参合率95.33%，万载正式启动实施新型农村社会养老保险试点。社会事业全面进步。市博物馆、图书馆、档案馆、城市规划展示馆和宜春日报社新楼顺利开工。科技入园工作主要经验在全省推广。教育“三名”工程扎实推进，新增省级重点中学6所，对全市119位名师、21位名校长、3所高中名校进行了表彰，上高被授予“全国推进义务教育均衡发展工作先进地区”。农村文化“一村一品”建设得到了中宣部、文化部领导的高度评价，万载得胜鼓荣获全国民间鼓乐大赛山花奖，宜春采茶歌舞剧院正式组建，大型现代采茶剧《村民小组长》在全省获奖，县（市、区）图书馆、文化馆维修改造和乡镇综合文化站建设工作有力推进，新建农家书屋680家。《宜春市志》编纂工作顺利完成初审和复审。基层医疗服务体系建设不断加强，全面启动了4所县级医疗标准化建设、16所中心卫生院加强型建设、4所城市社区卫生服务中心（站）的基础设施建设，完成村卫生所达标建设450个、中心乡镇卫生院10个，完成率均达100%。甲型H1N1流感防控工作措施得力,有效控制了疫情。体育工作成绩斐然，夺得第十一届全运会金牌3枚，占全省金牌总数的近1/3，获省政府表彰；成功举办市第三届运动会。人口计划执行良好，低生育水平进一步稳定，出生人口性别比趋于正常，利益导向全面落实，县站乡所国债项目顺利实施，宜丰、上高、奉新通过全国计划生育优质服务先进县复评。“万村千乡市场工程”有力推进，“家电下乡”工作名列全省前茅。电话“村村通”工程圆满完成。全面加强社会治安综合治理，深入推进和谐平安建设，全市治安状况进一步好转，被评为全省社会治安综合治理优秀市。精心部署“扫黑除恶”专项斗争，共铲除黑社会性质犯罪组织1个，恶势力团伙48个，特别是摧毁了中心城区两个作恶多年的特大涉黑涉恶犯罪团伙，人民群众拍手称快。高度重视信访化解工作，深入排查矛盾纠纷，积极开展“信访积案化解年”活动，清理和解决了一大批信访积案。深入开展安全生产专项整治，全市发生各类事故起数、死亡人数分别下降52.9%和7.2%，水上交通连续22年未发生死亡事故。

以“解放思想，迎战困难，加速赶超，争先进位”为主题，认真开展深入学习实践科学发展观活动，进一步增强了科学发展的自觉性和坚定性。强力推进政府自身建设，认真组织开展“机关效能年”活动。构建了市县乡三级行政服务体系，乡镇便民服务中心建设开创全省先河。在全省率先全面启动市县乡三级统一的电子政务办公平台建设，深入推进政府信息公开，努力提高行政效率和公共服务水平。严格实行公务购车经费、会议经费、公务接待经费、党政机关出国（境）经费预算“四个负增长”；各类行政审批事项压缩50%以上，整体承诺平均办结时限缩短47.8%。坚持向人大报告政府工作，加强与政协联系，认真接受人大及其常委会的法律监督和政协的民主监督，78件人大代表建议、161件政协委员提案全部办复，满意率达98.7%。依法行政工作积极推进，“五五”普法深入开展，法律援助和法律服务效能继续提升。认真落实党风廉政建设责任制，反腐倡廉建设进一步加强，公共资源交易管理体制有效理顺，交易活动进一步规范，执法监察、廉政监察、效能监察和审计监督成效明显。国防后备力量和民兵预备役建设、人民防空、国家安全、外事侨务、民族宗教、涉台事务、气象服务、防震减灾、妇女儿童、统计、档案、扶贫、老龄、残疾人和红十字会等各项工作都取得了新进步。

各位代表，回顾过去一年的工作，我们深感成绩的取得，是省委、省政府和市委正确领导的结果，是市人大依法监督、市政协民主监督以及社会各界大力支持的结果，是全市人民同心协力、迎难奋进的结果。在此，我代表市人民政府，向全市广大工人、农民、知识分子、干部和历任老领导、老同志，向各民主党派、工商联、无党派和社会各界人士，向驻宜部队、武警官兵、公安干警、中央和省驻宜单位，致以崇高的敬意！向所有关心、支持宜春发展的同志们、朋友们和海内外友好人士，表示衷心的感谢！

在看到成绩的同时，我们也清醒认识到，经济社会发展中依然存在一些突出矛盾和问题，主要表现在：经济增速与赶超目标仍有差距；经济结构性矛盾较突出，产业结构优化升级的任务还很艰巨，经济增长的资源环境约束仍在加剧；城乡和区域发展不平衡，城市基础设施承载能力尚需提高；社会矛盾依然凸显，社会稳定的压力依然较大，突发事件应急处理能力有待加强；农业抗御自然灾害和市场风险的能力较弱；就业和再就业压力较大，特别是农民工和大学毕业生就业问题突出，增加群众收入尤其是农民持续增收的难度加大；改革攻坚的任务艰巨，政府职能转变和作风建设必须进一步加强，等等。对这些问题，我们将以更有力的举措予以克服和解决。

二、2010年政府工作安排

2010年是实施“十一五”规划的冲刺年，是我市“止后移、争进位、站前列”的关键年。今年政府工作的总体要求是：全面贯彻党的十七大和十七届三中、四中全会精神，以邓小平理论和“三个代表”重要思想为指导，深入贯彻落实科学发展观，积极参与鄱阳湖生态经济区建设规划的实施，坚定不移地实施赶超式发展战略，坚定不移地把扩大固定资产投资规模作为加快发展的总抓手，千方百计推进重大产业和重大项目建设，千方百计推进结构调整和科技创新，千方百计加快宜居城市和小城镇建设，千方百计加快生态建设和农业产业化建设，千方百计推进重点领域改革，千方百计改善民生和保持社会和谐稳定，努力实现经济社会发展的新跨越。

综合考虑各种因素，今年国民经济和社会发展主要目标建议为：生产总值增长11%；财政总收入增长18%，其中，地方财政收入增长18%；规模以上工业增加值增长18%；全社会固定资产投资增长35%；社会消费品零售总额增长15%；引进省外5000万元以上工业项目资金增长20%；实际利用外资增长8%；外贸出口增长8%；城镇化率达到42%以上；农民人均纯收入增长8%，城镇居民人均可支配收入增长8%；城镇登记失业率控制在4.5%以内，人口自然增长率控制在8‰以内。

为实现上述目标，政府将重点抓好以下七方面工作：（一）狠抓重大产业和重点项目建设，加速扩大投资规模及经济总量

全力推进产业招商。以“重大产业和重点项目推进年”活动为抓手，大力实施产业招商，精心策划一批项目，成功引进一批项目，加快推进一批项目，顺利落户一批项目，进一步加速产业集群，推进质量振兴战略，提升产业规模和水平，努力形成在国内乃至国际上有重大影响的特色产业，实现招商新突破和产业大发展。继续在境内外组织重大产业招商推介会或洽谈会，力争引进超亿元人民币或一千万美元以上重点项目100个，超5亿元人民币重点项目30个，引资总额达到200亿元人民币。继续深入开展产业基地和工业园区“五上”创评活动，力争全市国家级和省级产业基地完成主营业务收入430亿元，增长30%，实现利税60亿元，增长30%，其中，丰城煤电循环经济主营业务收入突破130亿元，高安建筑陶瓷达到100亿元，樟树盐化工、袁州医药、市本级机电、奉新纺织、上高鞋革、万载花炮突破30亿元，丰城精品陶瓷、宜丰竹木加工、万载有机食品突破15亿元。以加快产业基地发展为依托，力争全市工业园区实现主营业务收入833.9亿元，利税总额达到88.4亿元，税金总额达到41.2亿元，三项指标均增长25%。积极策应和融入鄱阳湖生态经济区建设，加快区域内丰城、樟树、高安三市的产业对接，打造特色亮点。

积极抓好重大基础设施项目。盯紧国家和省投资动态，进一步加大跑项争资工作力度，着力提高项目前期工作水平，全力争取国家和省在资金、项目和政策方面的支持，力争更多的项目落地、投资进资。进一步完善重点工程跟踪服务机制，力争更多的重点工程项目尽快开工、尽早发挥成效。加大扩大内需中央投资项目管理力度，确保所有扩大内需中央投资项目按要求全部开工建设。加快宜春明月山机场建设，开工建设靖安洪屏抽水蓄能电站。力争建成昌铜高速公路南昌—奉新（靖安）段，并启动奉新（靖安）—铜鼓段建设。力争建成高安建筑陶瓷产业基地铁路专线和樟树盐化工基地铁路专线一期工程。建设宜春（高安）国家建筑陶瓷质量检验检测中心。加快启动丰城铁路专线、丰电三期和樟树盐化工码头建设。全力配合杭长铁路客运专线建设。力争启动宜春铁路货场和火车站的搬迁改造工作。完成昌金高速宜春收费站亭改建。加快推进咸宜吉铁路前期工作，确保列入国家“十二五”铁路建设发展规划。

大力推进中心城区六大产业发展。深化“做大中心城、做强市本级”战略，加速推进“三大战役”，坚持高位推进和市区联动，强化责任到位与操作效能，进一步加快基础设施、物流平台、服务平台等相关配套建设，不断增强产业集聚能力。着力引进落户一批投资规模大、科技含量高和创税能力强的支柱型重大项目，积极创建特色产业基地。把建设“锂电新能源产业基地”作为重中之重，强力推进平台建设、项目建设、资源整合，既重招商，力争引进35个重大项目，特别是“航母级”龙头企业，更重落地，力争20户企业投产，签约企业80%以上开工，尽快“初具规模、显现形象”，尽早建成拥有锂电企业群、占据产业制高点的“亚洲锂都”。按照“发展提速、比重提高、结构提升”的要求，突出发展生产性服务业，巩固提升生活性服务业，大力发展公共性服务业，进一步增强城市活力，促进服务业与其他产业的互动发展。

（二）加快农业产业化和新农村建设，力争“三农”工作的新优势

大力提升农业产业化水平。做强主导产业。认真实施“双十双百双千工程”和国家新增千亿斤粮食工程，坚定不移地抓好粮食、竹木、畜禽等10个优势特色产业，重点做强粮食、畜禽、竹木产业，做大油茶、蔬菜、中药材产业，做优茶叶、苎麻、水果、有机农业产业，力争2010年全市年销售收入超亿元的重点龙头企业扩大到50家以上，农民专业合作社达到1000家。做实农业招商。依托农业主导产业、优势农产品和农业龙头企业，大力开展农业产业招商，确保涉农产业招商引资额占全市招商引资总额的20%以上，每个县（市、区）引进1～3个亿元以上农业招商项目。做优城郊农业。以基础较好的城郊为突破口，统筹规划，精心组织，大力推进农业示范区建设，努力把城郊农业示范区建成新品种、新技术的示范基地和培训基地，市民的观光休闲基地，城市“菜篮子”的供应基地。2010年，全市争取建立10个1000亩以上的城郊农业示范区。建立和完善农业科技推广体系、动植物疫病防控体系和农产品质量安全体系。

扎实推进新农村及基础设施建设。大力实施“五大工程”：一是全面开展“森林宜春”建设。紧紧抓住我省实施鄱阳湖生态经济区战略和我市建设“国家现代林业示范市”的契机，大力推进造林绿化“一大四小”工程建设，努力形成“生态系统完备、林业产业发达、森林文化繁荣、人居环境优美”的绿色发展体系。2010年，确保完成植树造林35.27万亩；创建绿色示范集镇53个、绿色示范村庄200个，建设省级城市森林（湿地）公园10个。二是扎实推进农村清洁工程。进一步加强领导，加大投入，落实责任，推广宜丰垃圾无害化处理模式，逐步建立农村环境整治管理的长效机制，为顺利实现“一年试点启动、两年面上推开、三五年大见成效”的总体目标奠定基础。三是扎实推进和谐小康家园示范村建设。继续以行政村为单位，以产业为特色，采取领导挂、部门联、企业帮的办法，努力打造一批产业特色鲜明、生态环境优美、配套功能完善、集中连片的农村新型社区。四是扎实推进水利工程建设。突出抓好水库除险加固、农村水利建设，解决农村安全饮水问题，加快水利项目前期工作，不断完善农村小型水利工程管理体制改革与乡级水务站建设。切实做好防汛抗旱工作。五是扎实推进农村路桥建设。加大农村公路建设力度，全面实现“村村通”水泥路。继续按照“县（市、区）长负总责、一桥一领导”的要求，推进农村渡改桥工程，抓好工程建设节点和项目资金配套工作，严格项目资金和工程质量管理，确保年底竣工通车率达到100%。

认真落实各项惠农政策和强农举措。扎实开展减轻农民负担专项治理工作，紧盯农民建房、农村中小学收费、“一事一议”收费等重点领域，保持对加重农民负担行为打击的高压态势，确保农民负担不反弹。不折不扣地落实各项涉农补贴，加大对农业农村项目的监督抽查力度，确保政策落实不走样、农民群众得实惠。采取发放培训券的形式，积极开展农民工技能培训和创业培训，大力实施“阳光工程”、“雨露计划”和“金蓝领”等农民教育培训工程，提高农民素质和增收致富能力。创新计划生育利益导向机制，全面落实计划生育家庭奖励扶助、独生子女伤残死亡特别扶助等九项规定政策。

（三）加大中心城及城镇群的建设力度，全面提升城镇化水平

进一步增强中心城的辐射带动功能。以执行《宜春市中心城总体规划（2008-2030年）》为契机，围绕“把宜春建设成中部地区最佳宜居城市”目标，全面推进“城在林中、水在城中、人在景中”的生态园林城市创建，继续大力实施“13579”绿色工程，巩固提高城市创建和城市管理成效，力争今年中心城在全省率先获得“省级生态园林城市”称号。加速推进人口倍增计划。继续抓好机场A线和B线道路、明月立交桥、卢洲大桥、宜春南路贯通、环城西路320国道改造、秀江东路延伸二期建设，尽快完善中心城区的基础框架和服务性功能设施；抓紧宜阳大厦和文化艺术中心的装修施工，确保2010年全部交付使用；加大旧城改造力度，开工建设锦绣大桥、明月大道，适时启动状元洲公园改造、沿河中路改造、秀江西路改造、卢洲路延伸等项目建设。

切实推进县城建设。坚持城乡统筹发展，精心组织市域城镇体系规划的编制工作，加快以宜春中心城为中心，以县市城区为次中心，以乡村集镇为接点的城镇群建设，推进城乡一体化进程。积极策应中心城创建生态园林城市活动，加大县城生态园林建设力度，已获得省级园林城市的县市要积极启动创建省级生态园林城市工作；正在创建省级园林城的，要争取申创成功。进一步做活经营城市文章，按照“做大县城、做强经济、提升老城区、拓展新城区”的发展思路，坚持“四个优先”，即：规划优先，大手笔、高档次做好县城规划，拉大城市框架，增强县城承载功能；拆迁安置优先，进一步加快城关地区危旧房拆迁改造和城中村改造；基础设施建设优先，加快城关地区路网建设；社会事业优先，不断完善城市功能，聚集人气，致力提升城市的档次和品位。

努力加快小城镇建设。以建制镇为重点，加快培育特色优势产业，加大空心村改造，增强服务“三农”能力，加快农村富余劳动力转移，努力使小城镇成为当地辐射和带动农村发展的消费中心、生产要素集散中心、“三农”服务中心、社会保障和公共服务中心。建立科学合理的村镇规划管理体制，坚持规划一张图、审批一支笔、建设一盘棋、配套一条龙，大力规范村镇农民建房行为，切实改变规划无人管理、实施难以到位、建设混乱无序的问题。注重典型引路、示范先行，优先发展资源、区位和人文等比较优势明显、产业支撑有力、经济活力较强的小城镇，形成具有鲜明特色的区域经济格局。促进投资主体的多元化，重点抓好道路、绿化、供排水、环卫等基础设施和学校、卫生院、敬老院、文化中心等公共设施建设，力争在城镇污水处理、农村垃圾处理、农村危房改造等方面实现新突破。稳步推进户籍制度改革，放宽城镇户籍限制，使在城镇稳定就业和居住的农民有序转变为城镇居民。进一步加大依法用地管理力度，既加大新增建设用地报批力度，又依法依规供地，努力实现全市“零问责”目标。

（四）推进科技创新和结构调整，切实转变经济发展方式

切实提高科技创新能力。大力实施科技创新“六个一”工程，促进产业转型升级，主攻锂电新能源、生物和新医药、机械电子、新材料等优势高新技术产业，以创新型企业为实施主体，以重大科技项目为主抓手，以建设科技创新平台、高新技术产业特色基地和优势创新团队为重要支撑，加大科技投入，大力提升自主创新能力，带动产业结构优化升级。尽快建立产学研协调推进机制，促进科技成果向现实生产力转化。不断加快科技孵化园建设，促进中小企业孵化成长。继续推进科技入园、科技进村和科技惠民工程，切实强化科技服务手段。积极开展对外科技合作与交流，鼓励和引导企业以自研、引进、联合开发等方式进行创新。创新人才引进机制，围绕重点产业发展的需要，有针对性地引进高新技术和高科技人才。切实抓好知识产权保护，全市专利申请量年增长20%以上。

大力促进节能减排。坚持生态立市、绿色发展战略，严格实施生态市规划，严把建设项目环保准入关，严格执行节能减排目标责任制度和环境影响评价制度，全面完成省下达的减排计划。加强环保监控，抓紧完成市污染源监控中心和全市重点污染企业自动在线监控系统建设。全面推进县（市）污水处理设施工程建设，加强管网配套建设，确保一年内运行负荷达到设计能力的60%，三年内达到100%。逐步淘汰能耗高、污染重、技术落后的企业，重点在煤炭、竹木、化工、建材等行业开展资源循环利用。全力促进工程减排，重点抓好丰城电厂发电机组脱硫工程。推进生态建设，年内新增1个省级生态县、5个省级环境优美乡镇。

注重培育和扩大消费热点。认真落实各项政策措施，精心培育消费热点，进一步扩大市场需求。突出抓好房地产业健康发展，促进住房消费。切实把国家、省下调减免有关住房交易税费等政策落到实处，把握调控力度，稳定市场预期。加快推进廉租住房、经济适用住房建设和城乡危房改造。加强房地产市场监管，防止房价大起大落。突出抓好扩大消费政策措施的落实，促进农村消费。认真落实家电下乡、农机下乡、摩托车下乡、家电汽车以旧换新等政策，积极推进“万村千乡”市场工程、“农超对接”、“双百”市场工程、“信息下乡”工程，加强销售网点、配送体系、售后服务和信息系统建设，切实做好补贴资金审核兑付工作。继续支持和推进第三代移动通信网络的建设和运营。突出抓好旅游业发展，促进旅游节庆消费。继续推进以明月山温泉风景名胜区为龙头的旅游升温战，加大旅游促销力度，加强配套设施建设，推动全市旅游业发展由“升温”转为“升级”，力争实现年接待游客800万人次，增长22%，旅游总收入突破60亿元，增长24%，其中，明月山温泉风景名胜区接待游客100万人次，增长33%；旅游综合收入达6亿元，增长20%。启动国家5A级景区创建工作。精心筹办好第四届月亮文化节，着力拉动节庆消费、会展消费，进一步打响“月亮之都”品牌。

（五）深化重点领域各项改革，努力增强经济发展的内生动力和活力

全面完成企业改革任务。严格按照“谁主管，谁负责”的原则，实行分级、分口负责，层层落实工作责任制。在完成职工身份置换、分流安置的基础上，将工作重心转移到资产处置和推动后续改革上，按时间节点要求，确保5月底前，全面完成全市国有工业企业改革；年底前，全面完成全市国有非工业企业改革。

稳妥开展医药卫生体制改革。加快推进基本医疗保障制度建设，规范实施51所乡镇中心卫生院基本药物制度试点工作，健全医疗卫生服务体系，大力促进基本公共卫生服务均等化。加强卫生服务能力建设，力争实现90%以上的医疗卫生机构基本达到国家规定建设标准，基本建立比较完善的医疗卫生服务体系。

积极推进政府机构改革和效能建设。站在讲政治、顾大局的高度，精心部署，严谨实施，确保按时间节点全面完成市、县两级政府机构改革。深入开展“创业服务年”活动，进一步转变机关作风，提升创业服务水平。加快电子政务建设，基本实现市县乡三级党政群机关无纸化办公。深入推进廉政阳光工程建设和行政审批制度“两集中、两到位”改革。开展网上行政审批和电子监察，进一步简化办事流程、压缩办事时限、提高办事效率，不断强化行政服务中心“一站式”功能。认真落实和完善重大项目审批“绿色通道”制度，严格执行超时默许和缺席默认制度。加强公共资源交易监督、规范管理，开展以“服务在窗口、满意在中心”为主题的标准化窗口创建，建立健全“双优”工作绩效考核机制，进一步规范乡镇便民服务中心和公共资源交易站建设。严格执行涉企收费“三零两减半”制度，严格入企检查准入，健全投诉受理、处理、督查和举报奖励制度，形成直查快处反应机制，真正做到“有报必查、有查必果、查必到人、查必到位”。

深入推进财政金融改革。大力推进事业单位绩效工资改革、公务卡制度改革试点和村级公益事业“一事一议”财政奖补政策改革试点。进一步拓宽融资渠道，加强对拟上市企业的优选培育，根据企业意愿，积极支持和帮助企业通过各种途径上市。争取华伍制动尽快上市，正邦化工完成辅导并向国家证监委上报上市材料，争取百神药业进入辅导期，更多的企业启动股改，基本形成上市一批、储备一批、辅导一批的梯次格局。继续加大异地融资力度，支持外埠金融机构在我市开展业务，在加强信贷合作的基础上，争取引进更多的外埠金融机构入驻宜春。推进中小企业担保机构规范发展，积极支持民间资本参与市场投资，力争设立小额贷款公司，为设立本土银行积极创造条件。

（六）努力改善民生和维护社会安全稳定，进一步提升人民群众的幸福感、安全感与社会和谐水平

进一步健全社会保障体系。坚持把促进就业和创业作为经济社会发展的优先目标，不断拓宽就业渠道，抓好就业培训工作，千方百计增加再就业。精心组织实施好“千企万岗”帮扶对接就业活动，重点帮助高校毕业生和返乡农民工就业创业，全力做好创建首批国家级创业型城市工作。继续按照“多缴多享受”的原则，扎实推进社会保险扩面征缴。积极指导万载县实施好新型农村社会养老保险试点工作。健全完善城乡居民最低生活保障制度和医疗救助制度，切实保障弱势群体的生存权益。进一步做好失地农民生活保障工作。突出抓好保障性住房建设，着力解决中低收入家庭住房困难问题。

协调促进社会各项事业发展。全面落实义务教育经费保障机制，积极推动化解城区中小学大班额。全力推进教育“三名”工程，积极开展第二轮素质教育示范县评估。继续支持宜春学院做强做大，大力推进宜春职业技术学院创建省级示范性高职院校工作，发挥高校服务全市经济社会发展的重要作用。推进京剧和采茶戏进中小学课堂试点工作，办好第二届青少年科技小发明竞赛。大力繁荣文化事业，加快发展文化产业，培养发展一批具有地方文化创意的文化企业，打造1～2个省级文化产业基地，加强县（市、区）图书馆、文化馆及乡镇综合文化站建设。全面贯彻落实国务院《全民健身条例》，全力做好省第十三届运动会的备战参赛工作。深入推进“广播村村响”工程，确保全市40%以上行政村完成“村村响”建设任务。继续抓好“光明·微笑”工程，全面完成对全市所有具备手术适用症的白内障和唇腭裂患者免费手术治疗。加大甲流等传染病防控力度，确保公共卫生安全。加强食品、药品、餐饮卫生监管，保证群众食品和用药安全。继续稳定低生育水平，强化流动人口计生服务与管理，加大党员干部违法生育查处和出生人口性别比整治力度，推进人口计生综合改革，全面提升人口计生整体工作水平。

高度重视安全稳定。健全完善信访工作长效机制，坚持各级领导接待群众来访、重大疑难信访事项包案处理等制度，加大矛盾纠纷排查调处力度，有效控制集体访、越级访等非正常上访和群体性事件发生。加强社会治安综合治理，不断推进社会管理创新，加快“天网工程”建设，深入开展社会治安整治专项行动，依法打击各类违法犯罪，促进治安秩序进一步好转。不断推进人民调解、司法调解、行政调解“三调联动”，加大法律援助工作力度。落实安全生产责任制，健全重大危险源监控和重大隐患排查消除机制，深入开展重点领域专项整治，加快推进企业安全生产标准化建设，加强安全生产应急救援体系建设，提高应急处理能力，确保全市安全形势平稳好转。

（七）进一步加强政府自身建设，切实提高赶超发展的推进能力和服务水平

面对新一年各种困难的考验和多种风险的挑战，更加凸显加强政府自身建设的重要与紧迫。我们必须把思想和行动统一到省委、省政府和市委的决策部署上来，切实提高科学发展、赶超进位的推进能力和服务水平，履行好政府的庄重使命与艰巨职责。

强化法治意识，倍加注重科学民主决策。坚持把依法行政贯穿于政府工作的各个领域和各个环节，不断提高决策的科学性、民主化和透明度，努力使政府各项决策的程序依法规范、过程民主公开、结果科学公正，主动接受人民代表大会及其常委会的依法监督和人民政协的民主监督。按照赶超发展的目标要求，依法、科学、认真地做好“十二五”规划的编制工作。

强化执行意识，切实提升实际操作效能。大力提高政府执行力和操作力，确保政令畅通，提高行政效率。不断激发干部干事创业的热情和求真务实的能力，按照“项目化、时间表、责任人”的工作机制，进一步完善政府工作目标责任制、岗位负责制，把重点项目完成情况纳入对各主管部门和各级政府的考核指标，对落实不力的人与事启动行政问责。

强化为民意识，时刻关心群众切身利益。牢固树立“让群众满意就是最大政绩”的观念，把群众呼声作为首要的信号与职责，深入群众，体察民情，认真解决好看病上学、就业安置、征地拆迁、土地纠纷、社会治安等群众最关心最现实的利益问题。加大政务信息公开，全面推行行政权力网上运行工作，切实增强政府的公信力。

强化廉政意识，始终保持清正廉洁作风。严格落实党风廉政建设责任制，健全工程建设、土地交易、产权交易、政府采购、医药购销、资金监管等重点领域的反腐倡廉长效机制。大力提倡艰苦奋斗，坚决制止铺张浪费，严格控制楼堂馆所修建，严格控制一般性开支。开展“惩防体系推进年”活动，加大违法违纪案件查办力度，努力创造清正、廉洁、高效、务实的政务环境。

各位代表，我们已迈进充满希望的新一年，开始了充满挑战的新征程。让我们在省委、省政府和市委的领导下，高举中国特色社会主义伟大旗帜，以邓小平理论和“三个代表”重要思想为指导，深入贯彻落实科学发展观，坚定信心，开拓进取，乘势而上，为实现宜春经济社会赶超式发展目标而努力奋斗！
